# Long-term Indian optic neuritis study (THE LION STUDY): clinical, imaging features and visual behavior of optic neuritis in Indian population over two decades

**DOI:** 10.3389/fopht.2025.1688001

**Published:** 2026-01-22

**Authors:** Selvakumar Ambika, Santhakumar Durgapriyadarshini, Shikha Talwar Bassi, K. Padma Lakshmi, Smita Praveen, Vidhya Dharani

**Affiliations:** Department of Neuro Ophthalmology, Sankara Nethralaya, Chennai, Tamil Nadu, India

**Keywords:** atypical, idiopathic, Indian, NMOSD, optic neuritis, multiple sclerosis

## Abstract

**Purpose:**

The study aims to understand the disease course of optic neuritis in the Indian population and analyze their demographic patterns, clinical features, and treatment responses over a period of two decades.

**Methods:**

We retrospectively reviewed the medical records of patients with optic neuritis (ON) who presented to the neuro-ophthalmology clinic of a tertiary eye center between 1997 and 2017. Clinical profiles, neuroimaging features, and post-treatment visual outcomes were analyzed.

**Results:**

A total of 406 eyes from 314 patients were included in this study. The mean age at presentation was 36.28 ± 12.58 years. Females were more commonly affected (N = 191patients, 60%). Unilateral involvement was noted in 70% (222 patients), and pain was associated with 63% (200 patients). The mean vision at presentation was 20/500 ± 20/158 (log MAR 1.39 ± 0.898). Optic disc edema was noted on presentation in 50% of patients (204 eyes). The mean vision post-treatment was 20/44 ± 20/91 (log MAR 0.34 ± 0.66). Recurrent optic neuritis was observed in 26.75% of patients (84 patients). Neuromyelitis optica antibody (NMO Ab) was positive in 19 patients, and myelin oligodendrocyte glycoprotein antibody (MOG Ab) was positive in one patient. Optic neuritis was of idiopathic etiology in 88.8% of patients, multiple sclerosis (MS) in 5.8%, and 4.8% had conversion to MS by 3years.

**Conclusion:**

This ON study group of the Indian population had more optic disc edema on presentation and visual outcomes similar to those of other Asian studies, unlike ONTT. Idiopathic ON and neuromyelitis optica spectrum disorder-optic neuritis (NMOSD-ON) were more common than MS optic neuritis (MS-ON) in this cohort population. Prospective long-term follow-up of these patients can reveal the natural disease course, neurological associations, and treatment outcomes.

## Introduction

Optic neuritis (ON) is an inflammation of the optic nerve that that can be of idiopathic, demyelinating or other etiologies. It can occur as an isolated event or alongside CNS demyelinating diseases, such as multiple sclerosis (MS), neuromyelitis optica spectrum disorder (NMOSD), and myelin oligodendrocyte glycoprotein antibody-associated disease (MOGAD). ON can also be associated with systemic autoimmune, granulomatous, or infectious conditions. The optic neuritis treatment trial (ONTT) is the most widely accepted treatment study to date in the literature (1). Understanding the disease course and management of optic neuritis (ON) has advanced since the discovery of seromarkers such as neuromyelitis optica antibody (NMO Ab) and myelin oligodendrocyte glycoprotein antibody (MOG Ab) (2). Even before these seromarkers were identified, Jain et al. (3) in 1985, reported that MS was less common in India. This study analyzed hospital data across India over three decades and found that the MS incidence was 0.05% in South India and 1.58% in North India, with NMO-ON incidence of 9.3%. In 2012, Pandit et al. (4) reported that 49% of patients with ON in a demyelinating registry developed MS. This study was performed to enhance our understanding of ON behavior, its etiological associations, and treatment responses in the Indian population.

## Material and methods

Medical records of 314 patients with ON who presented to the neuro-ophthalmology services of a single tertiary care eye center in India between January 1997 and December 2017 were retrospectively analyzed. Patients with ON, with or without central nervous system (CNS) demyelination and with or without a previous history of ON, who presented within 6 weeks of the onset of vision loss and with a minimum of one post-treatment follow-up were included. Patients who presented beyond 6 weeks of onset of vision loss, who were lost to follow-up post-treatment, and those with optic neuropathies due to ischemic, hereditary, infiltrative, toxic, and traumatic etiologies or if associated with other ocular conditions like uveitis, glaucoma, corneal, or retinal pathology, were excluded. Snellen’s best-corrected visual acuity was converted to the logarithm of minimal angle of resolution (logMAR) for statistical analysis. Color vision, pupil, and anterior and posterior segment findings were noted. Humphrey visual field 30–2 details were noted when available. Serological assessment—basic hemogram, venereal disease research laboratory test (VDRL)/*Treponema pallidum* hemagglutination assay (TPHA), QuantiFERON TB Gold, rheumatoid arthritis factor (RA factor), antinuclear antibody (ANA), antinuclear cytoplasmic antibody (ANCA), angiotensin-converting enzyme (ACE), lysozyme, NMO Ab, MOG Ab, and Mantoux skin test details were recorded. NMO Ab testing was performed using an enzyme-linked immunosorbent assay (ELISA) for patients who presented between 2012 and 2014; subsequently, it was performed using a commercially available fixed cell-based indirect immunofluorescence assay (CIIFA). The MOG Ab test using a fixed cell-based assay was accessible only after 2017; hence, patients with ON who presented after this timeline underwent both NMOSD and MOGAD seromarker assessments.

MRI of the brain and orbit was performed with a 1.5 Tesla HDxt 16 channel GE system, mostly in a single referral radiology center; hence, standard imaging protocols such as fat suppression/short tau inversion recovery (STIR)/fluid-attenuated inversion recovery (FLAIR) were used for imaging the brain and orbit. Brain and orbital MRI were available for 297/314 patients. Imaging features such as optic nerve thickening, T2 hyperintense signals in the optic nerve and brain, location of the signal, and post-contrast enhancement were recorded. Whole spine MRI screening was performed in 21 patients. All patients were referred to a neurologist following neuro ophthalmic evaluation to neurologist for further assessment, CSF analysis, and treatment. The patients were subsequently reviewed for ophthalmic evaluation. All patients received intravenous methylprednisolone 1 g/day for 3–5 days, followed by oral steroids 1 mg/kg/body weight in weekly taper as acute management. Patients with poor visual recovery at 2–4 weeks received second-line treatments. By 2012, when the NMO Ab test was accessible at our center, there was more clarity in the management of patients with poor vision recovery, after first-line treatment. Intravenous immunoglobulin (IVIG) 0.4 mg/kg/day for 5 days was the treatment of choice by neurologists in the early decade of the study. Subsequently, rituximab was the common second-line therapy after acute management (1 g intravenous infusion in two doses, 2 weeks apart).

Petzold et al. (5) have formulated a criteria to diagnose optic neuritis as definite ON, and possible ON. According to this recommendation, the clinical criteria for ON diagnosis are subacute, monocular or binocular, painful or painless vision loss with optic nerve function defects. Paraclinical criteria for ON diagnosis include optic nerve or sheath enhancement or increased T2 hyperintense signals within the nerve on brain and orbital MRI. OCT of the optic nerve head showing optic disc edema or inter-eye difference in ganglion cell layer thickness of >4 μm or retinal nerve fiber layer of >5 μm, or presence of a seromarker NMO Ab, MOG Ab, or oligoclonal bands in CSF. According to these recent criteria, these clinical and para-clinical details were used to classify this cohort as either definite or possible ON. In the absence of paraclinical tests, a diagnosis of possible optic neuritis was made based on clinical findings. Patients with definite ON had clinical features with at least one para-clinical test. For atypical presentations like (painless and binocular) two paraclinical tests were used to categorize them as definite ON. Patients were diagnosed and classified as MS and NMOSD as per the revised McDonalds criteria and International Panel for NMO Diagnosis (IPND), respectively (6–8). Patients received disease-modifying therapy and immunosuppression as per the recommendation of the referral neurologist. Patients with recurrent ON and those who did not have clear imaging and CNS criteria for MS/NMOSD were refrained from medications such as interferons.

### Statistical methods

Descriptive statistics were computed for continuous variables, and frequency distribution was used to validate the distribution of categorical variables. An independent sample t-test was used for mean comparison between the two groups, and a paired t-test was used for pre-treatment and post-treatment comparison. The chi-square test for independence was used to determine the association between two qualitative variables. Binary logistic regression was used to determine the risk factors for the outcome variables. All statistical tests were performed using SPSS 23.0, and p-values less than 0.05 were considered statistically significant.

## Results

### Clinical characteristics

This study included 314 patients (406 eyes). The demographic and clinical characteristics of patients are presented in **Table 1**. Reduced color vision was observed in 209 eyes, and data were not available for the rest. Visual evoked potential (VEP) data were available for 207 eyes in this study. Flash VEP followed by conventional pattern reversal VEP for 1° and one-fourth degree checker sizes was performed. VEP responses showed extinguished waveforms in 25 eyes (6.16%) and delayed latency with reduced amplitude in 182 eyes (87%). Humphrey visual field analysis was performed in 231 patients. Generalized depression (124 eyes) was the most common field defect, followed by central or cecocentral defects (76 eyes) and altitudinal defects (60). Neurological features such as numbness, tingling sensations, and hemiparesis were noted in 10 patients (3.2%), and one patient had bladder incontinence due to myelitis.

**Table 1:** Baseline demographics and clinical characteristics.

Characteristics	% of patients(pts)
Demographics	
Female	68.8% (P = 0.0002)
Male	39.2%
Mean age (years)	36.28 ± 12.58 (P<0.001)
Ethnicity	
Northern India	0.96%
Southern India	52.9%
Eastern India	33.76%
Western India	2%
Central India	10%
North eastern India	0.32%
Clinical characteristics	
Mean vision on presentation	20/500 ± 20/158 (log MAR 1.39 ± 0.898)
Pain (N = 200 eyes)	63.7%
Laterality	
Unilateral (N = 222 pts)	70.7%
Bilateral (N = 92 pts)	29.3%
Disc appearance on presentation	
Retrobulbar neuritis (N = 94 eyes)	23.16%
Disc edema (N = 204 eyes)	50.2%
Optic atrophy (N = 108 eyes)	26.6%

### Diagnostic procedures and laboratory investigations

Brain and orbital MRI were available for 297 patients, of whom 280 underwent post-contrast imaging. MRI spine screening was performed in 21 patients. **Table 2** enumerates the imaging characteristics of patients with ON. Cerebrospinal fluid (CSF) analysis was performed on 43 patients. CSF analysis was normal in 28 patients (8.92%), nine patients (2.87%) had elevated protein levels, and six patients (1.91%) had oligoclonal bands. Blood test results were documented for 143 patients; 25 patients had elevated ESR, and seven patients had elevated C-reactive protein levels. The rheumatoid arthritis (RA) factor was positive in three patients, and the anti-nuclear antibody was positive in six patients. Angiotensin-converting enzyme levels were elevated in 17 patients, and serum lysozyme levels were elevated in three patients. One patient was positive for TPHA but negative for rapid plasma regain (RPR). The Mantoux skin test was positive in three patients, and the QuantiFERON TB Gold test was positive in one patient.

**Table 2:** MRI characteristics of optic neuritis patients in LION study.

MRI N = 297 patients	Characteristics	Patients (%)
Orbit—signals	T2 Hyperintense signals	245 (78.03%)
Contrast study N = 280 patients	Enhancement and thickening—Perineuritis	56 (20%)
	Thinning of optic nerve	6 (1.91%)
Segment of the optic nerve involved	Anterior Posterior	157 (64.08%) 61 (24.90%)
Brain—Signals	Present Absent	67 (21.34%) 230 (73.24%)
Location of signals	Juxta cortical	32
	Periventrcular	17
	Infratentorial	17
	Supratentorial	5
Spine—Signals N = 21patients	Present	11 (52%)

Details of optic nerve signals were not available = 46 patients.

Details of optic nerve segment involvement unavailable= 20 patients.

NMO Ab was performed in 156 patients, of whom 19 patients were NMO Ab-positive. **Table 3** shows the timeline of the tests and techniques used to detect NMO Ab. Among the patients who tested positive, nine were tested using the ELISA technique for NMO Ab and 10 were tested using the fixed CBA (CIIFA) technique for NMO Ab. NMO Ab was positive in CIIFA for these nine patients who tested positive with ELISA. Patients with a positive RA factor and elevated angiotensin-converting enzyme/serum lysozyme levels did not have any clinical features suggestive of autoimmune or granulomatous diseases, according to the referring immunologist and infectious disease consultants. Hence, this finding was considered clinically insignificant. Patients with positive ANA were noted to have low titers and did not have any associated systemic vasculitis, except for one patient who had a high ANA titer (1:1,280) and was also noted positive for NMO Ab. MOG Ab was performed in five patients. One patient with relapsing optic neuritis was incidentally positive for TPHA but negative for RPR. Subsequently, the patient was also positive for MOG Ab.

**Table 3:** Timeline of techniques used for detecting NMO Ab in study center.

Year	Technique
Before 2012	NMO Ab test not accessible
2012–2014	NMO Ab—*ELISA technique
2014 onwards	NMO Ab—*CIIFA technique

*ELISA, enzyme linked immunosorbent assay; *CIIFA, cell-based indirect immunofluorescence assay.

### Treatment and clinical course

ON patients were noted as definite and possible ON as per Petzold et al. criteria in Lancet Neurology (5).

Based on the clinical and paraclinical criteria suggested by the Lancet group, 206 patients of 314 were categorized as definite ON and 108 as possible ON. **Table 4** enumerates the mean visual outcomes analyzed in both groups. The p-value was not statistically significant (0.71) between the definite and possible ON groups. Of the 314 patients with optic neuritis, 264 (84%) had idiopathic ON, 18 (5.8%) had MS-ON, 19 (6.1%) had seropositive NMOSD-ON, four (1.3%) had seronegative NMOSD-ON, and one (0.3%) had MOG Ab optic neuritis (MOG-ON). A large subset of patients in this cohort did not undergo NMO Ab (158 patients) and MOG Ab (309 patients) testing, as the study included patients before the widespread availability of seromarker testing. Therefore, these patients were reviewed for possible NMOSD-ON and MOG-ON based on core clinical, imaging, and supportive features (7, 8). Possible NMOSD-ON features were observed in eight patients (2.6%) who did not undergo NMO Ab testing, and 85 patients (27%) had possible MOG-ON features who were not tested for MOG Ab.

**Table 4:** Mean visual outcome in last follow up in our cohort.

Group	Sample (N = 314)	Visual outcome in last follow-up
Total ON cohort	314	20/44 ± 20/91 (log MAR 0.34 ± 0.66)
Definite ON	206	20/50 ± 20/100 (log MAR 0.4 ± 0.7)
Possible ON	108	20/50 ± 20/80 (log MAR 0.4 ± 0.6)

P value—0.71.

At presentation, 18 patients were on oral steroids, three were on immunomodulator therapy (interferon beta 1a), and four were on immunosuppressants (azathioprine and mycophenolate mofetil). However, all patients received intravenous methylprednisolone 1 g/day for 3–5 days, followed by a weekly taper of oral steroids. One patient received intravenous immunoglobulin therapy, and four received rituximab. In this cohort, 20 MS suspect patients were on disease-modifying therapy (DMT), 16 patients were on interferon beta 1a (30 μg–40 μg once a week taken through the subcutaneous route), and four patients were on dimethyl fumarate (240 mg twice daily taken orally). Oral immunosuppressive therapy was administered for 34 patients, of which 24 patients were on azathioprine (75 mg/day–100 mg/day), one patient was on mycophenolate mofetil (500 mg/day), and 10 patients were on low-dose steroids (5 mg/day–15 mg/day). The mean vision post-treatment at the final follow-up was 20/44 ± 20/91 (log MAR 0.34 ± 0.66, P =<0.05). The mean follow-up was 2.03 years ± 3.02  years, and 84 patients had 3 years follow-up. Of the 314 patients, 84 (26.75%) had recurrent optic neuritis. By 3 years, four patients (4.8%) had MS conversion.

## Discussion

**Figure 1:**
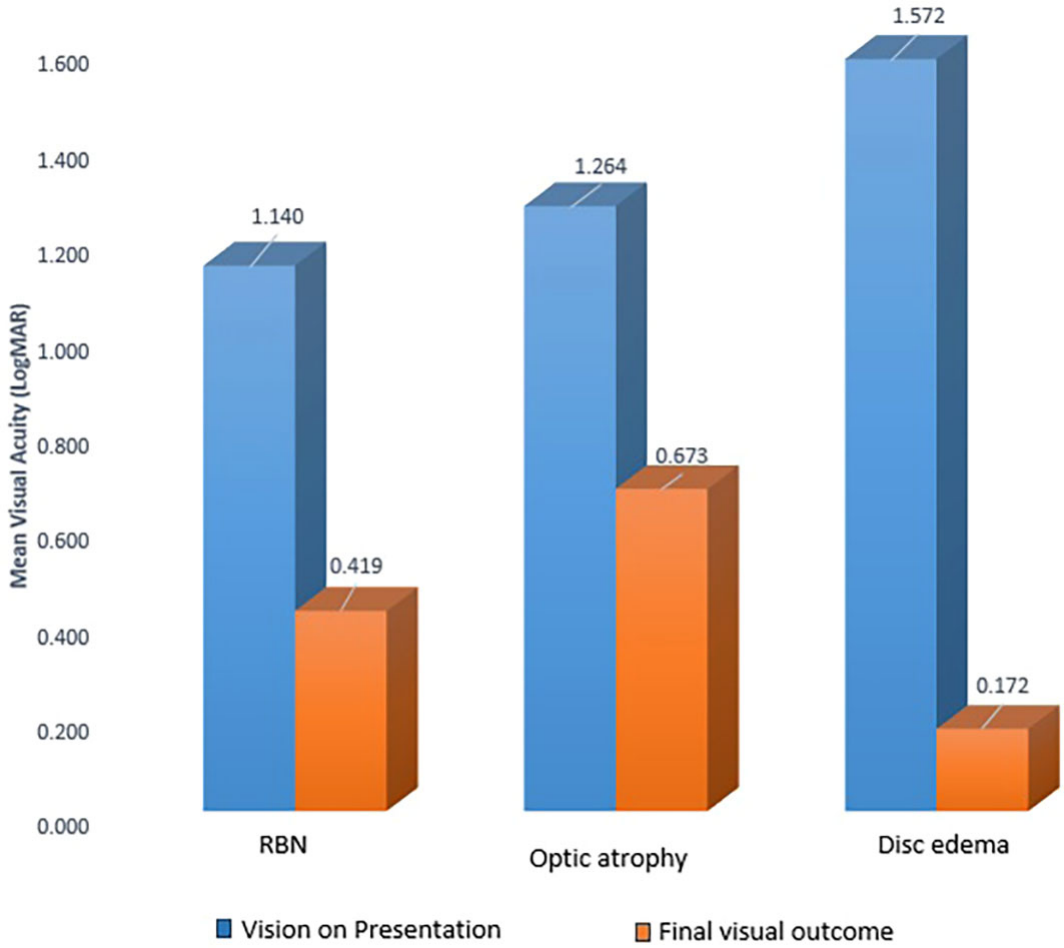
Bar diagram showing comparison of visual outcome in patients with retro bulbar optic neuritis (RBN), optic atrophy, and disc edema on presentation.

**Figure 2:**
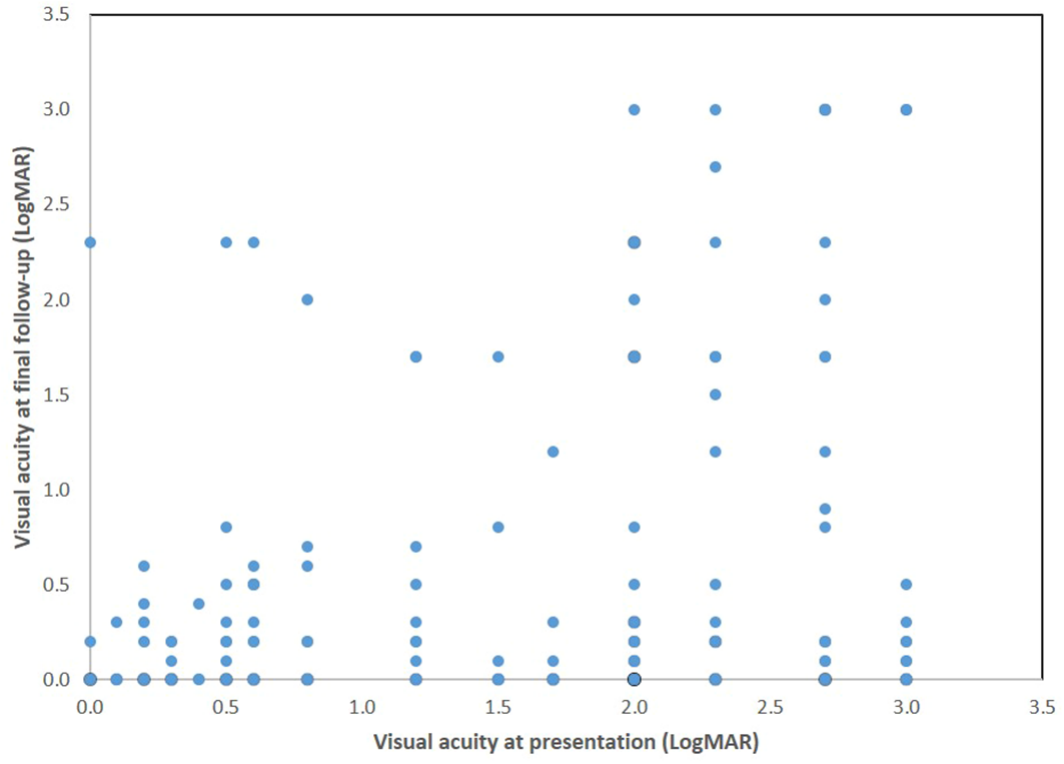
Scatter plot showing visual acuity at presentation of patients with optic neuritis and visual outcome at final follow-up.

The Optic Neuritis Treatment Trial (ONTT) (1) is the gold standard for the treatment of optic neuritis. However, the ONTT cohort does not have a diverse ethnic background, with hardly any Indian or Asian representation. In 1985, a hospital-based survey by Jain et al. (3) noted that MS was uncommon in India. A population-based survey from Mangalore noted the prevalence of MS and NMOSD to be 8.3/100,000 and 2.6/100,000, respectively (9). This cohort has a diverse Indian population, with the majority from the southern, eastern, and central parts of India. The mean age of presentation was third to fourth decade which is similar to other studies (9–12). Ishikawa et al. (13) noted NMOSD in Asian population affects mostly females than males but MOGAD affects both sexes equally which was similar to our results (14). The present study had less data on MOG-ON, probably because of the lack of access to MOG Ab during the study period. This study cohort had a high unilateral presentation and less pain, similar to other Asian studies (14–16). Approximately 50% of ON cases had disc edema on presentation, unlike in the West, where retrobulbar neuritis (64%) was more common. Irrespective of disc appearance, the mean vision loss at presentation was profound (20/500 ± 20/158). However, post-treatment vision at the final follow-up was better in patients with disc edema at presentation than in those with retrobulbar neuritis and optic atrophy (**Figure 1**). The mean post-treatment vision at the final follow-up was better than 20/50 (log MAR 0.4) in 78.4% of the patients, which was statistically significant (**Figure 2**). Although visual outcome was lesser than ONTT, it is comparable with other Asian studies (17–19). **Table 5** compares the demographics and clinical characteristics of patients with optic neuritis in the present study with those in other studies (15, 19–24).

**Table 5:** Comparison of demographics and clinical characteristics of optic neuritis in present study with other studies.

Study results	ONTT (19)	Taiwan (15)	Thailand (24)	LION study (Present study)
Year	1988–2006	1986–2003	2010–2020	1997–2017
Sample size pts (eyes)	448 (448)	109 (147)	171 (226)	314 (406)
Study design	Prospective	Retrospective	Retrospective	Retrospective
Age (yrs)	31.8	41.2	45	36.28
Female (%)	77.2	53.2	78.4	60.8
Pain (%)	92.2	58.7	51.5	63.7
Disc edema (%)	35.3	53.2	33.3	50.2
MRI brain signals (%)	48.7	34.9	NA	21.3
Recurrence (%)	NA	33.9	NA	26.7
Visual outcome logMAR >0.5 (6/18 in Snellen chart) (%)	92	NA	NA	78.4

The presence of T2 hyperintense brain signals in the periventricular region and white matter lesions on MRI brain and orbit have been associated with a high risk of conversion to MS (20). In the ONTT, 48.7% of patients had brain signals on presentation. In the ONTT, 25% of patients with a baseline normal MRI and 72% of patients with one or more brain lesions developed MS (20–22). Wakakura et al. (10) from Japan and Lim et al. (16) from Singapore reported 14% and 34% incidence of brain signals on presentation in their cohort, respectively. According to Lim et al. (16) and Lin et al. (15), the conversion rates to MS were 9.1% by 2 years and 14.3% by 5 years, respectively. In our study, 21.3% of patients (n = 67) had brain lesions on presentation, of which 4.8% developed MS by 3 years. As per ONTT, patients with more disc edema and less pain have less chance of conversion to MS which is similar to our study and other Asian studies (15, 22, 23). Our results are similar to those of Jain et al. (3), who reported that NMOSD is more prevalent than MS in India. Although this study cohort had less MOG Ab testing, we presume the possibility of MOG-ON can be higher in our population, as 27% of patients had clinical features of MOG-ON despite unknown serostatus. Long-term follow-up of this cohort can reveal more details.

From 1997 to 2007, patients with poor vision recovery following acute management were re-evaluated by a neurologist and then considered for the next line of therapy. Intravenous immunoglobulin (IVIG) was the second-line treatment administered by neurologists. Although blood tests were not part of the ONTT protocol, being an endemic zone for a few infectious diseases like TB, blood tests for granulomatous and infectious causes were performed even in those days, prior to immunosuppression. By 2012–2015, when NMO Ab testing was accessible to our center, there was more clarity in management patterns for poor vision recovery patients post-first-line treatment. Rituximab was the most common second-line therapy following acute management, irrespective of serological status. In our cohort, one patient received IVIG and four received rituximab. Treatment was challenging in patients with isolated ON and poor vision recovery, without imaging/neurological evidence for MS/NMOSD with negative or unknown serological status.

Univariate regression analysis was performed to analyze the risk factors associated with visual outcomes (**Table 6**). Patients with optic atrophy, painless attacks, extinguished waveforms on VEP, T2 hyperintense signal in the posterior segment of the optic nerve on brain and orbital MRI, positive NMO Ab, and recurrent attacks had poor treatment response and visual outcomes. Patients presenting with disc edema, painful, and monophasic attacks had better visual outcomes. These predictive factors are similar to those reported in the recent literature (25–28).

**Table 6:** Logistic regression analysis of risk factors influencing visual outcome.

Visual acuity better than 0.5 logMAR (6/18 in Snellen chart)		95% Confidence interval		
Variable	Odds ratio	Lower limit	Upper limit	P-value
Pain	2	1.12	3.59	0.02
Disc edema	2.98	1.43	6.22	0.004
Increased RNFL thickness	25.5	1.72	37.79	0.02
Monophasic	2.37	1.39	4.05	0.002
Visual acuity worse than counting fingers (CF)		95% Confidence interval		
Variable	Odds ratio	Lower limit	Upper limit	P value
Painless	2	1.12	3.59	0.02
Optic atrophy	2.32	1.22	4.43	0.01
Extinguished VEP	10.09	3.21	31.68	0
Recurrent	2.37	1.39	4.05	0.002
Positive NMO Ab	3.53	1.66	7.52	0.001

The limitations of this study are its retrospective nature, lack of control groups, and limited follow-up period. Seromarkers were not available for all patients, leading to a lack of details regarding the association between the NMOSD and MOGAD spectrum. Future prospective studies with long-term follow-up, including these details, are required in the Indian/Asian population to understand the natural disease course of ON in this part of the world.

## Conclusion

This ON cohort with a diverse Indian population presented with more disc edema, less pain, and less association with MS. Idiopathic ON was the most common followed by NMOSD-ON, and the visual outcomes were similar to those reported in other Asian studies, unlike the ONTT. Seromarker assessment is mandatory for the management and prognosis of optic neuritis. It is also important in this ethnic background, to rule out associated infective causes prior to long-term immunosuppression. Considering the higher incidence of NMOSD-ON, it is important to be more assertive in treating ON without classical neurological signs and an unknown serological status. This group must be refrained from receiving immunomodulatory therapy, such as interferons, to avoid further worsening of the disease. MOG Ab testing was performed in the later phase of this study. Therefore, there is a possibility of having higher MOG-ON in this cohort, which future studies can unveil. Hopefully, more prospective trials will help us understand ON behavior in India. It is unclear how long and how severe these patients need maintenance immunomodulatory agents. This poses a huge financial burden on patients in countries like India.

## Data Availability

The original contributions presented in the study are included in the article/supplementary material. Further inquiries can be directed to the corresponding author.
